# Bruxism in Attention Deficit Hyperactivity Disorder

**DOI:** 10.1192/j.eurpsy.2026.11497

**Published:** 2026-07-31

**Authors:** G. A. Unal

**Affiliations:** Psychiatry, Mersin Sehir Egitim ve Arastirma Hastanesi, Mersin, Türkiye

## Abstract

**Introduction:**

Bruxism is a masticatory muscle activity characterized by clenching and/or grinding of the teeth without a functional purpose, which can occur during sleep and while awake. The prevalence of awake bruxism is thought to be 22.1% to 31%, while sleep bruxism is thought to be 12.8%. Bruxism can lead to problems such as fractures and abrasions in teeth, loss of support and mobility in periodontal tissues, pain in the chewing system and orofacial region, and temporomandibular joint dysfunction. It is known that bruxism is associated with mental disorders, anxiety and intensity of stress. Bruxism has been reported to be significantly more common in children with attention deficit hyperactivity disorder (ADHD). There is an association between bruxism and ADHD, but studies on ADHHD and bruxism in adults are uncommon.

**Objectives:**

The aim of the study was to evaluate whether generalized orofacial pain complaints such as teeth grinding, jaw pain, jaw clicking, and headache were more common in those diagnosed with ADHD and/or taking psychostimulant medications.

**Methods:**

This study was conducted in the psychiatry clinic of a city education and research hospital between October 2024 and January 2025. A cross-sectional study was conducted on adults over the age of 18 diagnosed with ADHD who applied to the psychiatry outpatient clinic. Forty three participants met the eligibility criteria were included in this study. ADHD diagnose was based on the criteria of DSM-5. Self-report and clinical examination were used for bruxism. The presence and severity of temporomandibular joints disorder in the participants were evaluated using the Fonseca Anamnestic Index (FAI). The presence of bruxism in the participants was examined using a standardized assessment form (Standardized Tool for the Assessment of Bruxism-STAB Axis A).

**Results:**

The sample had a mean age of 32.7 years, of which 68% were male and 32% were female. Sleep bruxism and awake bruxism were evident in 51.2% and 58.1% respectively.

**Conclusions:**

Since bruxism is statistically significantly associated with ADHD, further studies on this subject would be useful. Awareness of ADHD symptoms in cases of bruxism will increase compliance with treatment and thus reduce orthodontic complications. Therefore, increasing awareness of the relationship between bruxism and ADHD will provide a multidisciplinary approach to treatment.

**Disclosure of Interest:**

None Declared

